# Book Notices

**Published:** 1863-07

**Authors:** 


### Book Notices.

A Practical Handbook of Medical Chemistry. By John E. Bowman, F.C.S., formerly Professor of Practical Chemistry in King's College, London. Edited by Charles L. Bloxam, Professor of Practical Chemistry in King's College, London. Third American from the Fourth and Revised London Edition, with Illustrations. Philadelphia: Blanchard & Lea. 1863.

The Medical Student's Vade-Mecum. A compendium of Anatomy, Physiology, Chemistry, Poisons, Materia Medica, Pharmacy, Surgery, Obstetrics, Practical Medicine, Diseases of the Skin, &c., &c. By George Mendenhall, M.D., Professor of Obstetrics and Diseases of Women and Children in the Medical College of Ohio, Member of the American Medical Association, &c., &c. Seventh Edition, Revised and Enlarged, with 224 Illustrations. Philadelphia: Lindsay & Blakiston. 1863.

A Practical Treatise on Fractures and Dislocations. By Frank Hastings Hamilton, A.B., A.M., M.D.; Lieutenant-Colonel; Medical Inspector of U. S. A.; Professor of Military Surgery and Hygiene, and of Fractures and Dislocations, in Bellevue Hospital Medical College; one of the Surgeons to the Bellevue Hospital, New York; Professor of Military Surgery, &c., in the Long Island College Hospital, Brooklyn; author of a Treatise on Military Surgery. Second Edition, Revised and Improved. Illustrated with 285 Woodcuts. Philadelphia: Blanchard & Lea. 1863.

The Action of Medicines in the System: Being the Prize Essay to which the Medical Society of London awarded the Fothergillian Gold Medal for 1852. By Frederick William Headland, M.D., B.A., F.L.S., Licentiate of the Royal College of Physicians, &c., &c. Fourth American Edition. Philadelphia: Lindsay & Blakiston. 1863.

A Theoretical and Practical Treatise on Midwifery, including the Diseases of Pregnancy and Parturition, and the Attentions required by the Child from Birth to the Period of Weaning. By P. Cazeaux, Member of the Imperial Academy of Medicine, Adjunct Professor in the Faculty of Medicine of Paris, Chevalier of the Legion of Honor, &c., &c., &c., &c. Adopted by the Superior Council of Public Instruction, and placed, by ministerial decision, in the rank of the Classical Works designed for the use of Midwife Students in the Maternity Hospital of Paris. Third American, translated from the Sixth French Edition. By Wm. R. Bullock, M.D.; with 140 Illustrations. Philadelphia: Lindsay & Blakiston. 1863.

All the foregoing works are excellent treatises on the several branches set forth on their title pages, and by authors of deservedly high reputation. They are new editions of works which have been long enough before the profession to be well known and duly appreciated; hence, no extended notice of their contents is required. The publishers have also executed their part of the task well: the paper, type, illustrations, and binding being all of excellent quality. They may be found for sale at the Book Store of W. B. Keen & Co., Lake Street, Chicago.

